# FAMILY CAREGIVER FACTORS AND SUBSEQUENT EMERGENCY DEPARTMENT UTILIZATION AMONG OLDER ADULTS WITH DISABILITY

**DOI:** 10.1093/geroni/igz038.802

**Published:** 2019-11-08

**Authors:** Julia Burgdorf, John Mulcahy, Halima Amjad, Judith D Kasper, Kenneth Covinsky, Jennifer L Wolff

**Affiliations:** 1 Johns Hopkins Bloomberg School of Public Health, Baltimore, Maryland, United States; 2 Johns Hopkins University Bloomberg School of Public Health, Baltimore, Maryland, United States; 3 Johns Hopkins University School of Medicine, Baltimore, Maryland, United States; 4 University of California San Francisco, San Francisco, California, United States; 5 Johns Hopkins University, Maryland, United States

## Abstract

Community-living older adults with disability are frequent Emergency Department (ED) users and most rely on family caregiver support. However, no prior research has examined associations between caregiver characteristics and subsequent ED utilization among older adults. We draw on a sample of 2,521 community-living older adults with mobility/self-care disability and their primary family caregivers to identify caregiver characteristics associated with all-cause or potentially preventable ED use. We use Cox proportional hazards regression to separately model the likelihood of all-cause and potentially preventable ED use as a function of caregiver characteristics. Models account for competing risk of mortality and adjust for measures of older adults’ socio-demographic characteristics, health status, and survey wave. About half (52.5%) of older adults incurred 1+ ED visit and 26.8% incurred 1+ potentially preventable ED visit within 12 months of interview. Adjusting for survey wave and older adult sociodemographic characteristics and health status, older adults were at greater risk of all-cause ED use if their primary caregiver provided greater than 40 hours of care per week (HR: 1.22, 95% CI: 1.04-1.43; p=0.02), helped with health care tasks (HR: 1.26; 95% CI: 1.08-1.46; p<0.01), or experienced physical strain (HR: 1.18; 95% CI: 1.03-1.36; p=0.02). Older adults were at greater risk of potentially preventable ED use if their primary caregiver helped with health care tasks (HR: 1.25; 95% CI: 1.02-1.54; p=0.03). Findings highlight the relevance of caregiver factors to older adults’ ED use and suggest the need for assessment and support of family caregivers in the care delivery setting.

